# Music-based therapeutic interventions for medical school students with emotional regulation and mental health: a pre-post cohort study

**DOI:** 10.3389/fpsyg.2024.1401129

**Published:** 2024-05-30

**Authors:** Quan Chen, Chaoqin Mao, Laihua Qi, Yang Luo, Guangyao Yang, Lei Wang, Chen Liu, Chuansheng Zheng, Jinxiang Zhang, Cheng Fan

**Affiliations:** ^1^Department of Radiology, Union Hospital, Tongji Medical College, Huazhong University of Science and Technology, Wuhan, China; ^2^Hubei Province Key Laboratory of Molecular Imaging, Wuhan, China; ^3^Department of Rehabilitation, Union Hospital, Tongji Medical College, Huazhong University of Science and Technology, Wuhan, China; ^4^Union Hospital, Tongji Medical College, Huazhong University of Science and Technology, Wuhan, China; ^5^Department of Thoracic Surgery, Union Hospital, Tongji Medical College, Huazhong University of Science and Technology, Wuhan, China; ^6^Department of Emergency Surgery, Union Hospital, Tongji Medical College, Huazhong University of Science and Technology, Wuhan, China; ^7^Department of Geriatrics, Union Hospital, Tongji Medical College, Huazhong University of Science and Technology, Wuhan, China

**Keywords:** music therapy, medical students, depression, anxiety, SCL-90

## Abstract

**Purpose:**

Depression and anxiety are prevalent mental health challenges among college students. Music therapy has shown effectiveness in addressing depressive symptoms and enhancing psychosomatic functioning. This study aimed to evaluate the effectiveness of a 4-step structured music therapy program in improving mood and reducing symptoms of depression and anxiety among medical school students.

**Materials and methods:**

The self-controlled study involved 45 medical school students (21 men and 24 women) aged 18–24 years to examine the prevalence of depression and anxiety, common mental health issues among medical school students. Participants underwent psychological assessment using the Symptom Checklist-90 (SCL-90), Self-Rating Anxiety Scale (SAS), and Self-Rating Depression Scale (SDS). An 8-week music therapy intervention, comprising four steps—sociality, interaction, music lessons, and creative expression—was administered.

**Results:**

Before-intervention, 55.6% and 15.6% students were identified as suffering from depression and anxiety respectively. Post-intervention, significant reductions in psychological distress, particularly in the Global Severity Index (GSI) and Positive Symptom Total (PST) on the SCL-90 scale, were observed (*P* < 0.05). Male students exhibited notable improvements in various psychological symptoms compared to females. Junior grade students demonstrated greater improvements, and clinical medicine students exhibited significant enhancements in specific areas post-intervention.

**Conclusion:**

The structured music therapy program showed promising results in improving mood and regulating emotions among medical school students. Music therapy holds potential as a holistic approach to address mental health challenges in this demographic.

## 1 Introduction

Depression and anxiety are prevalent mental health challenges among college students, with global statistics ranking them fourth and sixth, respectively, in the major burdens faced by individuals aged 10–24 years (Global Burden of Disease Study and Injuries, 2020). These disorders significantly contribute to self-harm and injury, particularly among adolescents and young adults (Li et al., 2023). Notably, research indicates that depression is a primary emotional concern for college students, particularly those in university settings (Pratt and Brody, 2014; Rotenstein et al., 2016). The prevalence of common mental health issues, including depression and anxiety, has been on the rise among college students in recent years (Lattie et al., 2019; Li et al., 2023). Notably, medical school students, due to the prolonged duration required for acquiring knowledge and skills in the field of medicine, face heightened levels of mental and physical stress (Adams, 2004; O'Rourke et al., 2010; Quek et al., 2019; Zeng et al., 2019). Various studies consistently show that medical school students experience a higher prevalence of depression and anxiety compared to the general population (Adams, 2004; Dyrbye et al., 2006; Khan et al., 2006; Ahmed et al., 2009; Hope and Henderson, 2014; Tabalipa et al., 2015; Azad et al., 2017; Quek et al., 2019).

Music therapy, recognized as the professional use of music and its elements for therapeutic purposes, has emerged as a promising intervention in medical, educational, and everyday settings (Maratos et al., 2008). It utilizes musical activities to stimulate individuals and elicit physical responses, with the goal of enhancing health through tools such as music, relationships, and reflection. Music therapy is comprised of active and receptive approaches that also integrate verbal processing. These approaches include various ways of engaging with music, including receptive, improvisational, compositional, and recreative (Bruscia, 2014). Research involving patients diagnosed with mental disorders has consistently shown significant improvements in mental health following interventions that utilize music as a primary tool (Edwards, 2006; Mössler et al., 2011; Rebecchini, 2021; Zhang et al., 2022). Additionally, numerous studies have highlighted the multifaceted benefits of music, ranging from enhancements physical and mental wellbeing (Fancourt et al., 2014; Khan et al., 2018; Wang and Agius, 2018). However, the potential of music therapy to improve emotional wellbeing, alleviate depression, or reduce anxiety among medical school students—a unique demographic group—remains largely unexplored. In this study, our aim was to address the psychological challenges encountered by medical school students through the use of comprehensive screening tools, including the SCL-90, SDS, and SAS scales. Our primary objective was to evaluate the efficacy of music therapy as a non-pharmacological intervention in enhancing mood and alleviating symptoms of depression and anxiety among medical school students.

By specifically targeting this demographic group, our research seeks to fill a crucial gap in the literature and provide valuable insights into the effectiveness of music therapy as a tailored intervention for improving the mental health and wellbeing of medical school students. The integration of music therapy into mental health support strategies for this population shows promise and merits further investigation.

## 2 Materials and methods

### 2.1 Participants

**Figure 1:**
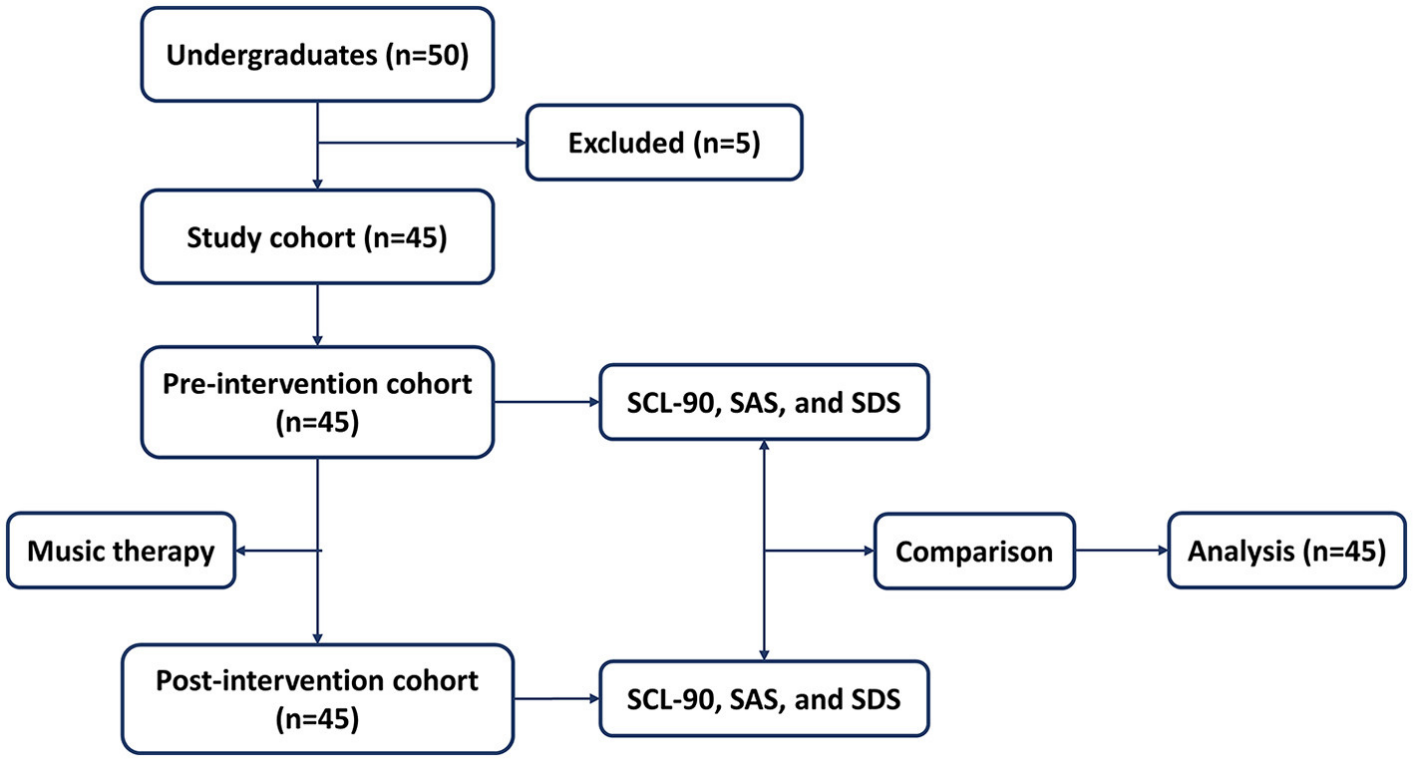
Workflow of study participants.

The research, conducted at Tongji Medical College, Huazhong University of Science and Technology in Wuhan, Hubei, China, spanned from April to November 2023 and received ethical approval from the Ethics Committee of Tongji Medical College. This collaborative effort involved three trained music therapists, two clinical doctors, and facilitators who are educators at the medical college. The study focused on medical school students, with inclusion criteria comprising individuals aged between 18 and 24 years. The investigation aimed to assess the efficacy of scales (SCL-90, SAS, and SDS) before and after music therapy. Exclusion criteria encompassed immediate suicidal thoughts or behaviors, a history or current presence of mental illnesses, and concurrent music or antidepressant therapy elsewhere. The participant workflow is elucidated in Figure 1.

### 2.2 Music therapy intervention

**Figure 2:**
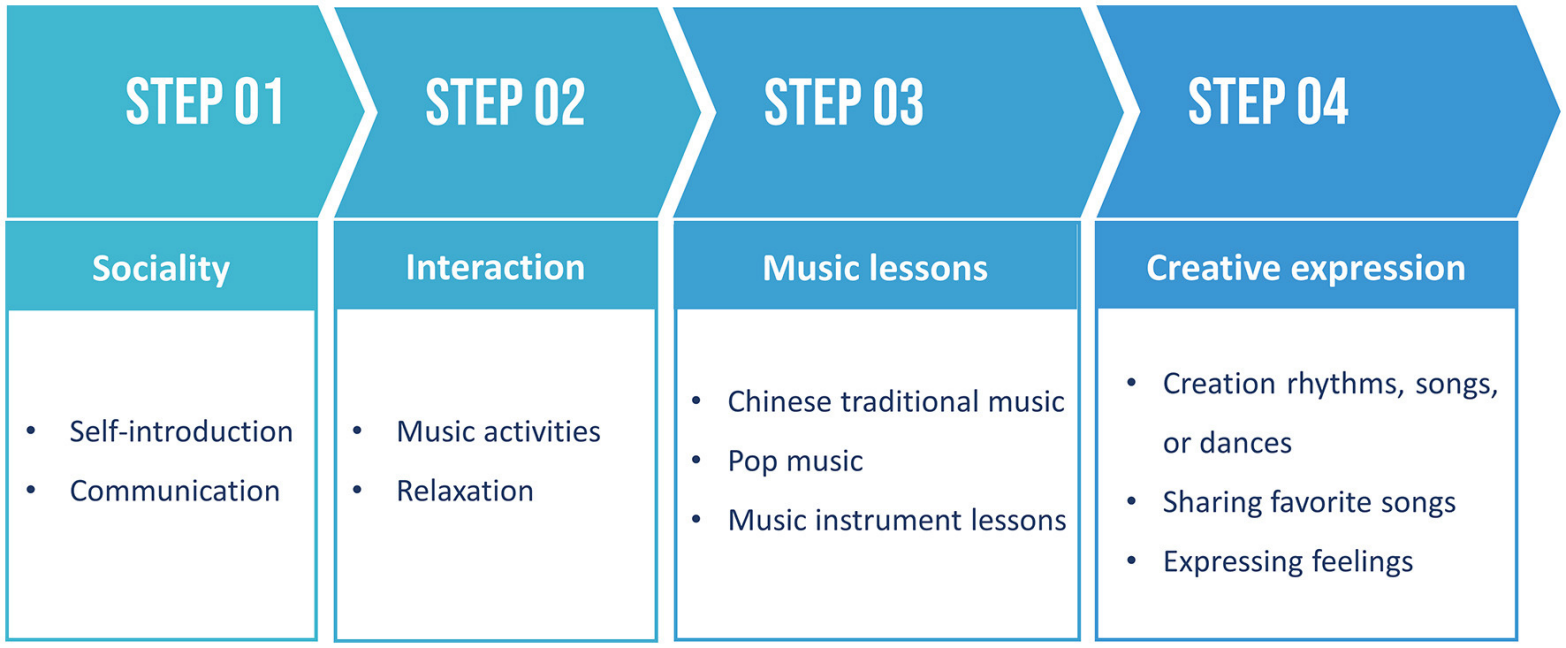
Music therapy protocol.

A total of 8 weekly music therapy sessions, each lasting 2 h, were implemented. The music therapy protocol involved four sequential steps outlined in Figure 2.

Music therapy interventions were structured as follows:

Step 1: social interaction

- Introduction: This step served as an icebreaker, introducing participants to the concept of music therapy and facilitating self-introductions among group members to foster familiarity.
- Communication: Participants were encouraged to share their experiences, technical knowledge, social skills, music backgrounds, or emotions. This facilitated exploration of individuality and self-expression within the group dynamic.

Step 2: interactive music activities

- Music Activities: Participants engaged in various music-related group activities such as listening to music, playing musical instruments, singing, rhythmic interventions, and dancing. These activities helped participants identify emotions, connect mind and body through music, alleviate social anxiety, and promote self-expression.
- Mediation and Progressive Relaxation: Directed listening sessions guided participants through mediation, progressive relaxation, or breathing exercises, aiding in emotional processing and relaxation (Gallego-Gómez et al., 2020; Hwang et al., 2023).

Step 3: Music lessons

- Chinese Traditional Music: Participants received lessons on Chinese pentatonic songs, which are familiar to Chinese students and are known for their calming and mood-stabilizing effects. These lessons aimed to improve self-esteem and overall wellbeing (Zhang and Gao, 2022; Fu and Tu, 2023; Wu et al., 2023).
- Popular music: Lessons encompassed various genres including folk, jazz, blues, reggae, funk, pop, and rock, catering to diverse musical preferences. Popular music, known for its widespread acceptance and catchy lyrics, was included to promote relaxation and emotional awareness, contributing positively to mental health (Kulinski et al., 2022; Zhang and Hu, 2023).
- Music instrument lessons: rhythm instruments (drums, cymbals, gongs, etc.) and melody instruments (guitar, piano, guzheng, dulcimer, erhu, flute, etc.).

Step 4: creative expression

- Creating and sharing: Participants engaged in creative expression through rhythm creation, songwriting, dance improvisation, and musical improvisation. This allowed them to express their feelings through music, fostering cognitive health and wellbeing (Dingle et al., 2021).
- Sharing favorites: Participants shared their favorite songs or melodies, discussing their meanings and enhancing social connections within the group, thus uplifting mood and fostering a sense of community (Dingle et al., 2021).

### 2.3 Scales

This study employed the SCL-90, SDS, and SAS to screen and evaluate psychological disorders, depression, and anxiety in medical school students. Validity and reliability studies of the SCL-90, SAS, and SDS scales yielded good psychometric qualities (Yu et al., 2019; Wang et al., 2022). In our study, Cronbach's α values were found to be above 0.97, 0.78, and 0.88, respectively.

The SCL-90, a self-report questionnaire, gauges psychological symptoms experienced in the past 7 days. Each of its 90 items is rated on a five-point scale (0. Normal; 1. Mild; 2. Moderate; 3. Severe; 4. Extreme), contributing to nine scores across major symptom dimensions: Somatization, Obsession-Compulsion, Interpersonal Sensitivity, Depression, Anxiety, Hostility, Phobic Anxiety, Paranoid Ideation, Psychoticism, and Additional items. Additionally, three global indices—Global Severity Index (GSI), Positive Symptom Total (PST), and Positive Symptom Distress Index (PSDI)—can be calculated (Holi et al., 1998; Schauenburg and Strack, 1999; Schmitz et al., 2000; Maremmani et al., 2010, 2018; Dang et al., 2020).

The SDS and SAS, commonly used scales for assessing depression and anxiety (Dunstan et al., 2017; Dunstan and Scott, 2020). each comprise 20 items with raw scores ranging from 20 to 80. Questions are rated on a 1–4 Likert scale, from “a little of the time” to “most of the time.” SDS scores categorize depression levels: 20–44 (normal); 45–59 (mild depression); 60–69 (moderate depression); 70 and above (severe depression). SAS scores categorize anxiety levels: 20–44 (normal range); 45–59 (mild to moderate anxiety); 60–74 (severe anxiety); 75 and above (extreme anxiety) (Zung, 1971, 1974; Guo and Huang, 2021).

### 2.4 Data analysis

All data were processed and analyzed utilizing SPSS 26.0 software. Data normality was evaluated using the Shapiro-Wilk test in SPSS. As a result of non-normality, the paired Wilcoxon signed-rank test was utilized to assess differences in psychological indexes between cohorts. Count data were analyzed using the χ^2^ test. A significance level of *P* < 0.05 was considered indicative of statistical significance.

## 3 Results

### 3.1 Participant characteristics

The study included 45 medical school students, comprising 21 males and 24 females, who were divided into two groups: the pre-music therapy cohort and the post-music therapy cohort. Table 1 presents the participants' characteristics, including age, gender, grade, and major.

**Table 1:** Participant characteristics.

**Characteristics**	**Students (%) (*n =* 45)**
**Gender**	
Male	24 (53)
Female	21 (47)
Age, years (Mean ± SD)	20.76 ± 1.19
**Grade**	
≤ 2	25 (56)
>2	20 (44)
**Major**	
Clinical	23 (51)
Others	22 (49)

### 3.2 Music therapy helps to improve mood and regulate emotions

Before and after music therapy, the enrolled students' scores on the SCL-90, SDS, and SAS scales were recorded. Initially, the mean values for GSI, PST, and PSDI were 0.39, 24.0, and 1.47, respectively. Of the total 45 students, 25 were identified as suffering from depression, accounting for 55.6%, while 7 students (15.6%) were in a state of anxiety (Table 2).

**Table 2:** Participant characteristics of the SCL-90, SAS, and SDS scales among medical students in the pre- and post-intervention cohorts.

**Scales**	**Pre-intervention**	**Post-intervention**	**Statistical value**	***P***
**SCL-90, mean** ±**SD**				
GSI	0.39 (0.22, 0.81)	0.30 (0.13, 0.59)	−2.265^a^	0.024^*^
PST	24.0 (15.0, 42.0)	20.0 (9.5, 36.0)	−2.693^a^	0.007^**^
PSDI	1.47 (1.13, 1.71)	1.37 (1.04, 1.67)	−0.572^a^	0.567
**SDS, n (%)**			0.241^b^	0.971
normal	20 (44)	21 (47)		
mild depression	20 (44)	18 (40)		
moderate depression	4 (9)	5 (11)		
severe depression	1 (2)	1 (2)		
**SAS**, **n (%)**			1.077^b^	0.584
normal	38 (84)	38 (84)		
mild to moderate anxiety	6 (13)	7 (16)		
severe anxiety	1 (2)	0		
extreme anxiety	0 (0)	0		

Mean (P25, P75); ^a^z value; ^b^χ^2^ value; ^*^p < 0.05; ^**^p < 0.01.

Post-intervention, a significant decrease in GSI (*P* = 0.024) and PST (*P* = 0.007) was observed, indicating a lower overall psychological distress and reduced symptom severity. These findings suggest that music therapy effectively improved mood and may serve as a means to regulate emotions. However, there were no significant differences observed in SDS or SAS scores following the intervention.

### 3.3 Improvement of interpersonal sensibility and hostility after music therapy

Following music therapy intervention, the total score of SCL-90 significantly decreased compared to pre-intervention levels, while no notable difference was found in SAS or SDS scores post-intervention (Table 3). Further analysis of the nine subscales of SCL-90 revealed significant reductions in students' interpersonal sensibility and hostility (*P* = 0.007 and *P* = 0.005, respectively) post-intervention. Additionally, improvements in anxiety and paranoid ideation were observed, although they did not reach statistical significance at the 0.05 level.

**Table 3:** Comparison of SCL-90, SCL-90 subscale, SAS, and SDS of medical students in the pre- and post-intervention cohorts (*n* = 45).

**Scales**	**Pre-intervention**	**Post-intervention**	***Z***	***P***
SCL-90	33.0 (18.5, 72.0)	25.0 (11.0, 52.5)	−2.100	0.036^*^
Somatization	7.0 (4.0, 13.5)	6.0 (3.0, 11.5)	−1.752	0.080
Obsession-compulsion	5.0 (2.5, 10.0)	4.0 (1.0, 10.0)	−0.911	0.362
Interpersonal sensibility	4.0 (2.0, 9.0)	3.0 (1.0, 6.0)	−2.708	0.007^**^
Depression	5.0 (2.0, 11.0)	3.0 (1.0, 8.5)	−1.311	0.190
Anxiety	3.0 (1.0, 8.0)	2.0 (0.0, 4.5)	−1.907	0.057
Hostility	1.0 (0.0, 3.0)	1.0 (0.0, 2.0)	−2.835	0.005^**^
Phobic anxiety	2.0 (0.0, 4.5)	1.0 (0.0, 2.5)	−0.872	0.383
Paranoid ideation	1.0 (0.0, 2.0)	1.0 (0.0, 2.0)	−1.915	0.056
Psychoticism	2.0 (1.0, 5.0)	1.0 (0.0, 4.5)	−1.474	0.140
Additional items	3.0 (0.0, 4.0)	2.0 (0.0, 4.0)	−1.380	0.167
SAS	35.0 (31.9, 41.3)	35.0 (30.0, 41.9)	−0.954	0.340
SDS	46.3 (35.0, 53.8)	46.3 (35.0, 52.5)	−1.393	0.164

Mean (P25, P75); ^*^p < 0.05; ^**^p < 0.01.

### 3.4 Gender differences in music therapy

Regarding gender differences in SCL-90 and its subscales, SAS, and SDS, male students exhibited significant improvements in somatization, interpersonal sensibility, and hostility post-intervention (*P* = 0.04, *P* = 0.023, *P* = 0.021, respectively, Table 4). However, females showed no statistically significant differences in any subscales, SDS, or SAS. Paranoid ideation and SDS exhibited trends of improvement, but the *P*-values did not reach 0.05.

**Table 4:** Comparison of SCL-90, SCL-90 subscale, SAS, and SDS of medical students in the pre-intervention and post-intervention cohorts with gender (female and male).

**Scales**	**Male**				**Female**			
	**Pre-intervention**	**Post-intervention**	***Z***	***P***	**Pre-intervention**	**Post-intervention**	***Z***	***P***
SCL-90	42.0 (23.0, 74.0)	29.5 (14.3, 51.5)	−1.572	0.116	31.0 (11.5, 64.0)	16.0 (8.0, 60.5)	−1.200	0.230
Somatization	10.0 (4.3, 15.8)	6.5 (4.0, 13.5)	−2.050	0.040^*^	5.0 (2.5, 11.5)	5.0 (3.0, 10.5)	−0.285	0.776
Obsession-compulsion	6.0 (3.0, 11.5)	5.0 (2.3, 10.0)	−0.772	0.440	4.0 (1.5, 9.0)	3.0 (1.0, 9.5)	−0.416	0.678
Interpersonal sensibility	4.0 (3.0, 9.3)	2.0 (1.3, 5.8)	−2.267	0.023^*^	4.0 (1.0, 9.0)	3.0 (1.0, 6.5)	−1.531	0.126
Depression	6.5 (3.0, 12.0)	4.5 (1.0, 8.8)	−1.250	0.211	4.0 (0.0, 10.5)	2.0 (0.5, 9.0)	−0.524	0.600
Anxiety	3.0 (1.0, 7.0)	2.0 (0.3, 4.8)	−0.985	0.324	3.0 (0.5, 8.0)	1.0 (0.0, 4.5)	−1.542	0.123
Hostility	2.0 (1.0, 3.0)	1.0 (0.0, 2.0)	−2.303	0.021^*^	1.0 (0.0, 2.0)	0.0 (0.0, 2.0)	−1.663	0.096
Phobic Anxiety	2.0 (1.0, 4.5)	1.5 (0.0, 2.8)	−0.987	0.324	1.0 (0.0, 4.5)	1.0 (0.0, 2.5)	−0.213	0.831
Paranoid Ideation	1.0 (0.0, 2.0)	0.5 (0.0, 2.0)	−0.862	0.389	1.0 (0.0, 3.0)	1.0 (0.0, 1.5)	−1.805	0.071
Psychoticism	2.0 (1.0, 6.0)	1.0 (0.3, 4.8)	−0.931	0.352	2.0 (0.5, 4.0)	1.0 (0.0, 4.5)	−1.117	0.264
Additional items	2.5 (1.0, 4.0)	2.0 (1.0, 4.0)	−0.413	0.680	3.0 (0.0, 5.0)	2.0 (0.0, 5.0)	−1.583	0.113
SAS	36.9 (33.8, 41.3)	36.9 (30.6, 43.4)	−0.865	0.387	33.8 (28.8, 40.0)	31.3 (28.8, 39.4)	−0.280	0.779
SDS	46.9 (39.1, 53.4)	47.5 (36.6, 53.4)	−0.134	0.893	46.3 (33.1, 55.0)	40.0 (30.0, 52.5)	−1.823	0.068

Mean (P25, P75); ^*^p < 0.05; ^**^p < 0.01.

### 3.5 Junior grades showed significant improvements after music therapy

To assess the effects of music therapy on students at different grades, scales were analyzed for 25 junior ( ≤ 2) grade students and 20 senior (>2) grade students. Interestingly, junior students demonstrated significantly lower scores in SCL-90 total score (*P* = 0.0039), interpersonal sensibility (*P* = 0.015), hostility (*P* = 0.007), and paranoid ideation (*P* = 0.044) post-intervention. However, no significant differences were observed in the senior group after music therapy (Table 5). These results suggest that music therapy may be more effective or suitable for junior medical school students.

**Table 5:** Comparison of SCL-90, SCL-90 subscale, SAS, and SDS of medical students in the pre-intervention and post-intervention cohorts with grade ( ≤ 2 and >2).

**Scales**	≤ **2**				**>2**			
	**Pre-intervention**	**Post-intervention**	**Z**	**P**	**Pre-intervention**	**Post-intervention**	***Z***	***P***
SCL-90	48.0 (21.0, 78.0)	37.0 (9.0, 52.5)	−2.059	0.039^*^	28.0 (15.0, 49.0)	21.5 (11.3, 53.0)	−0.728	0.466
Somatization	10.0 (4.0, 17.0)	8.0 (3.0, 14.0)	−1.644	0.100	7.0 (4.0, 11.8)	5.0 (3.0, 7.0)	−0.786	0.432
Obsession-compulsion	6.0 (2.5, 10.0)	5.0 (1.5, 11.0)	−1.013	0.311	3.0 (2.3, 8.5)	3.5 (1.0, 7.8)	−0.459	0.646
Interpersonal sensibility	4.0 (3.0, 10.0)	3.0 (2.0, 5.0)	−2.423	0.015^*^	4.5 (1.3, 6.8)	3.0 (0.3, 7.0)	−1.368	0.171
Depression	7.0 (2.0, 12.0)	5.0 (1.0, 9.0)	−1.203	0.229	5.0 (2.0, 8.5)	2.0 (1.0, 8.3)	−0.617	0.537
Anxiety	3.0 (1.5, 9.5)	2.0 (0.0, 5.5)	−1.536	0.125	2.5 (0.3, 4.8)	1.5 (0.0, 3.8)	−1.088	0.277
Hostility	1.0 (0.0, 5.5)	1.0 (0.0, 2.0)	−2.710	0.007^**^	1.0 (0.0, 2.0)	1.0 (0.0, 2.0)	−0.811	0.417
Phobic anxiety	2.0 (0.5, 4.5)	1.0 (0.0, 2.0)	−1.209	0.227	2.0 (0.0, 4.5)	2.0 (0.3, 3.0)	−0.106	0.915
Paranoid ideation	1.0 (0.0, 3.0)	1.0 (0.0, 1.5)	−2.012	0.044^*^	1.0 (0.0, 1.8)	0.5 (0.0, 2.0)	−0.329	0.742
Psychoticism	3.0 (1.0, 6.0)	1.0 (0.0, 4.5)	−1.475	0.140	2.0 (0.0, 4.0)	1.5 (0.0, 4.8)	−0.096	0.923
Additional items	3.0 (1.5, 6.0)	2.0 (0.5, 5.0)	−1.859	0.063	1.5 (0.0, 4.0)	2.0 (0.0, 3.8)	−0.145	0.885
SAS	35.0 (30.6, 45.0)	35.0 (30.0, 43.8)	−1.023	0.306	35.0 (32.8, 39.4)	35.0 (29.1, 41.3)	−0.140	0.889
SDS	47.5 (36.3, 56.3)	46.3 (35.6, 58.1)	−1.542	0.123	45.6 (34.1, 53.4)	45.0 (33.1, 52.5)	−0.676	0.499

Mean (P25, P75); ^*^p < 0.05; ^**^p < 0.01.

### 3.6 Clinical medicine students exhibited significant improvements after music intervention

The enrolled students represented various healthcare majors, including Clinical Medicine, Pharmacy, Nursing, Radiology, Public Health, Stomatology, and Laboratory Medicine, with clinical medicine students comprising 51.0% of the total. A comparison of the effects of music therapy on clinical medicine students and those in other majors revealed significant improvements in hostility (*P* = 0.002), paranoid ideation (*P* = 0.037), and additional items (*P* = 0.004) among clinical medicine students, while no significant difference was observed in students from other majors post-intervention (Table 6).

**Table 6:** Comparison of SCL-90, SCL-90 subscale, SAS, and SDS of medical students in the pre-intervention and post-intervention cohorts with major (Clinical and Others).

**Scales**	**Clinical**				**Others**			
	**Pre-intervention**	**Post-intervention**	***Z***	***P***	**Pre-intervention**	**Post-intervention**	***Z***	***P***
SCL-90	49.0 (26.0, 82.0)	22.0 (11.0, 65.0)	−1.901	0.057	27.5 (14.8, 50.0)	27.5 (9.8, 47.8)	−0.779	0.436
Somatization	11.0 (5.0, 19.0)	6.0 (3.0, 13.0)	−2.486	0.013^*^	5.0 (3.0, 10.5)	5.5 (3.0, 10.3)	−0.357	0.721
Obsession-compulsion	6.0 (3.0, 10.0)	6.0 (2.0, 10.0)	−0.619	0.536	5.0 (1.8, 9.3)	3.0 (0.8, 8.8)	−0.587	0.557
Interpersonal sensibility	4.0 (1.0, 10.0)	3.0 (1.0, 5.0)	−1.820	0.069	4.0 (2.8, 6.3)	3.0 (1.8, 7.0)	−2.037	0.042^*^
Depression	7.0 (3.0, 13.0)	2.0 (1.0, 12.0)	−1.220	0.222	4.5 (1.8, 9.5)	3.5 (1.0, 8.3)	−0.655	0.513
Anxiety	3.0 (1.0, 11.0)	1.0 (0.0, 7.0)	−1.599	0.110	3.0 (0.0, 4.0)	2.0 (0.0, 3.3)	−1.010	0.312
Hostility	2.0 (0.0, 7.0)	1.0 (0.0, 3.0)	−3.035	0.002^**^	1.0 (0.0, 2.3)	1.0 (0.0, 2.0)	−0.329	0.742
Phobic anxiety	2.0 (0.0, 5.0)	1.0 (0.0, 2.0)	−1.057	0.291	1.0 (0.0, 3.3)	1.5 (0.0, 3.0)	−0.199	0.842
Paranoid ideation	1.0 (0.0, 4.0)	1.0 (0.0, 2.0)	−2.087	0.037^*^	0.5 (0.0, 2.0)	0.5 (0.0, 2.0)	−0.122	0.903
Psychoticism	3.0 (0.0, 7.0)	1.0 (0.0, 5.0)	−1.071	0.284	2.0 (1.0, 4.3)	1.5 (0.0, 4.0)	−1.301	0.193
Additional items	4.0 (2.0, 6.0)	2.0 (1.0, 5.0)	−2.844	0.004^**^	1.0 (0.0, 3.0)	1.5 (0.0, 4.0)	−0.812	0.417
SAS	35.0 (32.5, 45.0)	36.3 (30.0, 45.0)	−0.595	0.552	35.0 (28.8, 40.3)	33.8 (29.7, 41.3)	−0.447	0.655
SDS	47.5 (35.0, 57.5)	47.5 (36.3, 58.8)	−0.417	0.677	46.3 (33.4, 52.8)	45.6 (32.2, 51.3)	−1.378	0.168

Mean (P25, P75); ^*^p < 0.05; ^**^p < 0.01.

## 4 Discussion

This pioneering study is the first to utilize the SCL-90, SDS, and SAS scales to assess the impact of a 4-step structured music therapy program on medical school students. The results revealed promising outcomes, especially among male, junior grade, and clinical medicine students, indicating improvements in mood regulation and emotional wellbeing. This highlights the potential of music therapy as a non-pharmacological intervention for addressing mental health challenges among medical school students.

Medical school students are under immense academic, interpersonal, and emotional pressures, which often lead to heightened levels of anxiety and depression (Dyrbye et al., 2006; Hope and Henderson, 2014; Brenneisen Mayer et al., 2016). Our research found that the prevalence of depression and anxiety among Chinese medical school students was 55.6% and 15.6%, respectively, surpassing global rates (Brenneisen Mayer et al., 2016). While previous studies have shown the positive impact of music therapy on college students' mental wellbeing (Finnigan and Starr, 2010; Zhang et al., 2022; Finnerty et al., 2023; Nwokenna et al., 2023), no research has explored its efficacy specifically among medical school students. By focusing on this demographic, our study aims to bridge this gap in the literature and provide valuable insights into the effectiveness of music therapy as a tailored intervention.

In response, we designed an 8-week, 4-step music therapy program tailored specifically for medical school students. The program included various components aimed at fostering social interaction, engaging in music activities, learning music theory, and encouraging creative expression. Step 1 focused on fostering social connections through self-introduction and communication exercises, helping students acclimate to their environment, alleviate tension, and build trust with peers and instructors. Step 2 incorporated common music therapy techniques such as playing instruments, singing, and rhythmic interventions, along with progressive relaxation exercises to directly improve students' mental state, divert attention, and reduce tension and anxiety (Robb, 2000; Kim, 2008). Step 3 involved teaching students music theory, including different music genres, Chinese pentatonic songs, and various musical instruments. This aimed to enhance students' music appreciation skills and aesthetics, thereby improving subjective wellbeing and amplifying the therapeutic effects of music therapy (Robb, 2000; Che et al., 2022; Fu and Tu, 2023). The final step encouraged students to engage in creative expression by creating rhythms, songs, or dances through improvisation. This was followed by sharing favorite songs and expressing feelings, enabling students to understand and acknowledge their own emotional states and needs, express themselves freely, and build self-confidence, ultimately alleviating stress and anxiety. This comprehensive approach addresses various aspects of psychological wellbeing, providing medical school students with a holistic therapeutic experience tailored to their unique challenges and needs.

This study employed an innovative approach by utilizing three psychological scales—SCL-90, SDS, and SAS—to screen for mental health issues among medical school students. While various psychological scales, such as the Beck Depression Inventory (BDI) (Baldassin et al., 2008) and State-Trait Anxiety Inventory (STAI) (Du et al., 2022), are commonly used to assess depression and anxiety, they primarily focus on cognitive and affective aspects. In contrast, the combination of SCL-90, SDS, and SAS offers a more comprehensive understanding of psychological symptoms and provides a broader evaluation of depression and anxiety symptoms (Zung, 1971, 1974; Dunstan et al., 2017; Dunstan and Scott, 2020).

Using these methods, we observed a significant reduction in the GSI and PST on the SCL-90 scale following music therapy intervention. These findings indicate that music therapy may effectively improve mood and regulate emotions among medical school students. Notably, there were gender differences in response to music therapy, with male students showing significant improvements in somatization, interpersonal sensibility, and hostility compared to females. This suggests the need for gender-specific approaches in mental health interventions. Furthermore, the study explored the differential effects of music therapy based on academic grade and major. Junior grade students exhibited significant improvements across various scales post-intervention, consistent with previous research (Ludwig et al., 2015). Additionally, our investigation uncovered noteworthy insights into students majoring in clinical medicine, who demonstrated significant enhancements in hostility, paranoid ideation, and other factors compared to peers from different healthcare majors. These results emphasize the importance of tailoring music therapy interventions according to both academic progression and field of study, highlighting the nuanced nature of mental health interventions within academic contexts. Recognizing the unique needs and challenges faced by students in different healthcare disciplines is crucial for designing effective intervention strategies. By acknowledging these variations, healthcare professionals can develop targeted approaches to address specific issues and promote overall wellbeing among students pursuing diverse paths within the healthcare field. Furthermore, our findings suggest the potential benefits of integrating music therapy as a complementary intervention in clinical medicine education, potentially enhancing students' mental health and overall academic experience. Such integration could contribute to their professional development, fostering a healthier and more resilient future healthcare workforce.

While the results of this study are promising, several limitations should be acknowledged. The sample size was relatively small, and the study duration was limited to 8 weeks. Long-term follow-up and larger-scale studies would provide more robust evidence of the sustained effectiveness of music therapy in this context.

## 5 Conclusion

In conclusion, this study underscores the potential of music therapy as a non-pharmacological intervention for mitigating mental health challenges among medical school students. The structured music therapy protocol employed here demonstrated promising outcomes in enhancing mood and emotional regulation, notably among male students, those in junior grades, and pursuing clinical medicine. By introducing an alternative or complementary treatment approach, this research contributes to addressing the mental wellbeing of students, achieving its primary aim of mood improvement.

## Data availability statement

The raw data supporting the conclusions of this article will be made available by the authors, without undue reservation.

## Ethics statement

The studies involving humans were approved by the Ethics Committee of the Affiliated Union Hospital in Tongji Medical College of Huazhong University of Science and Technology (no.ChiCTR2200056141). The studies were conducted in accordance with the local legislation and institutional requirements. The participants provided their written informed consent to participate in this study. Written informed consent was obtained from the individual(s) for the publication of any potentially identifiable images or data included in this article.

## Author contributions

QC: Writing—original draft, Formal analysis, Data curation. CM: Writing—original draft, Data curation. LQ: Writing—original draft, Data curation. YL: Writing—original draft, Formal analysis, Data curation. GY: Writing—original draft, Validation, Software. LW: Writing—original draft, Investigation. CL: Writing—original draft, Resources. CZ: Writing—review & editing. JZ: Writing—review & editing. CF: Writing—review & editing, Project administration.
